# Voluntary Assisted Dying in Australia—Key Similarities and Points of Difference Concerning Eligibility Criteria in the Individual State Legislation

**DOI:** 10.1007/s11673-023-10228-9

**Published:** 2023-01-23

**Authors:** Michaela Estelle Okninski

**Affiliations:** Adelaide Law School, University of Adelaide, North Tce, Adelaide, SA 5005 Australia

**Keywords:** Voluntary assisted dying, Eligibility criteria, Australia

## Introduction

The legal landscape concerning voluntary assisted dying (“VAD”) has significantly changed in Australia. On November 29, 2017, the State Parliament of Victoria, Australia made headlines by becoming the first Australian state to pass VAD legislation (*Voluntary Assisted Dying Act 2017* (Vic) (“*VAD Act* (Vic)”). After an implementation period of about eighteen months, the legislation came into force on June 19, 2019 (Parliament of Victoria 2017), rendering Victoria the first state to legalize the somewhat controversial practice.^1^ Reflecting back, it is nearly impossible to conceive that over the next five years, every state in Australia would enact VAD legislation (*Voluntary Assisted Dying Act 2019* (WA), “*VAD Act* (WA)”; *End-of-Life Choices* (*Voluntary Assisted Dying*) *Act* 2021(Tas), “*VAD Act* (Tas)”; *Voluntary Assisted Dying Act 2021* (SA), “*VAD Act* (SA)”; *Voluntary Assisted Dying Act 2021* (Qld), “*VAD Act* (QLD)”; *Voluntary Assisted Dying Act 2022* (NSW), “*VAD Act* (NSW)”), and it cannot be denied that Victoria acted as the catalyst for the widespread law reform that ensued. More recently, on December 1, 2022, the Restoring Territory Right Bill 2022 (Cth) passed both houses of parliament restoring the Northern Territory and ACT’s legislative power to pass laws in relation to VAD by repealing the *Euthanasia Laws Act 1997* (Cth) which had been in force for over twenty-five years. The *Restoring Territory Rights Act 2022* (Cth) commenced operation on December 13, 2022.

Now that all Australian States have enacted VAD legislation, there is a broad consensus concerning some aspects of VAD in Australia, and there is evidence of a distinctive “Australian model,”^2^ especially in relation to defining eligibility criteria. Despite these similarities, interesting points of difference have emerged on the more complex issues such as conscientious objection of healthcare practitioners (see *VAD Act* [Vic] s 7(a)—(f); *VAD Act* [WA] s 9; Government of Western Australia 2019, 50–53; *VAD Act* [Qld] s 84), and the prohibition placed on healthcare practitioners from initiating discussions on VAD (see *VAD Act* [Vic] s 8; *VAD Act* [SA] s 12; *VAD Act* [Qld] s 7(1), (2); *VAD Act* [Tas] s 17; *VAD Ac*t [NSW] s 10).

The purpose of this Recent Developments is to briefly consider VAD legislation throughout Australia, drawing out key distinctions and points of similarity regarding the eligibility criteria in each separate Australian state. Due to the brevity of this article, it is not possible to engage in an in-depth analysis of the entire statutory response to VAD. Furthermore, the complexities surrounding the more contentious issues of conscientious objection and prohibition on initiating discussion on VAD cannot be considered within the confines of this discussion. It is also necessary to note that it is beyond the scope of this article to comment on whether a particular legislative approach operational in one state is preferable to another, as VAD is still in its infancy in Australia. Therefore, this article is limited to a selected overview of the statutory response to defining eligibility to request access to VAD, with specific focus on residency requirements and definition of the underlying medical condition.

## Overview of Eligibility Criteria

Voluntary assisted dying commenced operation in Victoria on June 19, 2019; in Western Australia on July 1, 2021; and in Tasmania on October 23, 2022 (Parliament of Victoria 2019; Government of Western Australia 2022; Tasmanian Government 2022). However, in January 2023, VAD will commence operation in Queensland and South Australia, and the *VAD Act* (NSW) is expected to commence operation on November 29, 2023 (Queensland Government 2022; Government of South Australia 2022; NSW Government 2022). Therefore, for the first time in Australian history, VAD will be operational in all Australian States by the end of 2023, giving eligible persons the right to request a hastened death.

### Eligibility Criteria

#### Age and Residency Requirements

There is consistency between the individual states concerning some aspects of the legislative approach to access VAD. For instance, access to VAD is restricted to persons aged eighteen years and over, who can demonstrate Australian citizenship or permanent residency status. Additionally, the person must establish that they have resided in that particular state for at least twelve months prior to making a first request for VAD (*VAD Act* (Vic) s 9(1)(a), (b); *VAD Act* (WA) s 16(1)(a), (b); *VAD Act* (Tas) ss 10(1)(a)(b), 11(1)(a)(i), (ii), (b); *VAD Act* (SA) s 26(1)(a), (b); *VAD Act* (Qld) s 10(1)(e), (f); *VAD Act*(NSW) s 16(1)(a), (b)(i),(ii)(c)). However, greater flexibility to this approach has been incorporated into the Tasmanian, Queensland, and New South Wales legislation in relation to residency requirements.

In these three states, persons who cannot satisfy the basic Australian citizenship or permanent residency requirements discussed above are permitted to request VAD if they can demonstrate that they have resided in Australia for at least three continuous years prior to making the first request (*VAD Act* (Tas) s 11(1)(a)(iii); *VAD Act* (Qld) s 10(1)(e)(iii); *VAD Act* (NSW) s 16(1)(b)(iii)). Further to this, the *VAD Act* (Qld) and the *VAD Act* (NSW) go one step further, permitting persons to apply for an exemption from the state residency requirements, conditional upon satisfying that they have a substantial connection to the State and “there are compassionate grounds for granting the exemption” (*VAD Act* (QLD) s 12 (1), (2); *VAD Act* (NSW) s 17).

Therefore, it is clear that there are differences concerning the legislative approach with regard to Australian citizenship and federal and state residency status. However, irrespective of these nuances, it is evident that the individual states have taken strict measures to circumvent the negative consequences associated with VAD tourism. The state residency requirements even operate to exclude interstate citizens and permanent residents of Australia from immediately accessing VAD, unless they can satisfy the residency exemptions incorporated into the Queensland and New South Wales legislation (see Parliament of Victoria 2017, 2948; Government of Western Australia 2019, 19–21; Queensland Law Reform Commission 2021, 35–36).^3^

#### Underlying Medical Condition

Each individual state legislation strictly prohibits persons from accessing VAD where mental illness or disability are the sole underlying medical condition (*VAD Act* (Vic) s 9(2), (3); *VAD Act* (WA) s 16(2); *VAD Act* (Tas) s 10(2)(a)(b); *VAD Act* (SA) s 26(2), (3); *VAD Act* (Qld) s 13; *VAD Act* (NSW) s 16(2)(a), (c)). For example, the *VAD Act* (Qld) provides that “it is declared that a person with a disability or mental illness … is not eligible [to access VAD] only because the person has the disability or mental illness” (s 13(1)(b)). However, it is necessary to note that persons with a mental illness or disability are not precluded from accessing VAD under the respective state legislation—they can, subject to meeting all the eligibility criteria (see for example, Queensland Law Reform Commission 2021, 31). The purpose of the mental illness and disability exclusionary provisions are to clarify that mental illness and disability *alone* are not recognized as underlying medical conditions for the purpose of the law.

A core condition of eligibility is that the person must be close to death in order to request access to VAD. The general legislative approach regarding life expectancy is that the underlying disease, illness, or medical condition (“the UMC”) be “expected to cause death within weeks or months, not exceeding 6 months,” or within twelve months if the UMC is neurodegenerative (*VAD Act* (Vic) s 9(1)(d)(iii), (4); *VAD Act* (WA) s 16(1)(c)(ii); *VAD Act* (Tas) s 6(1)(c)(i)(ii); *VAD Act* (SA) s 26(1)(d)(iii), (4); *VAD Act* (NSW) s 16(1)(d)(ii)(A), (B)). In Queensland the legislation does not establish a separate life expectancy requirement conditional upon whether the UMC is neurodegenerative or not and instead requires that death is expected to occur within twelve months or less (*VAD Act* (Qld) s 10(1)(a)(ii); Queensland Law Reform Commission 2021, 28–31). However, the legislative approach regarding life expectancy in Tasmania is unique when compared to the other Australian jurisdictions.

Tasmania have adopted a more patient-centred approach to this criterion and have included in the legislation an exemption to meeting the life expectancy requirement which applies “if the Commission is satisfied that the prognosis of the person’s relevant medical condition is such that the paragraph should not apply” (*VAD Act* (Tas) s 6(3), (4)). Thus, the Tasmanian position on life expectancy operates as a less arbitrary rule, permitting consideration of a person’s broader circumstances (University of Tasmania 2021, 35). In addition to the above criteria, diagnosis of an illness, disease, or medical condition that is advanced, progressive, and will cause death is mandatory in nearly all states, with the exception of Tasmania where the term “progressive” has been excluded (*VAD Act* (Vic) s9(1)(d)(ii); *VAD Act* (WA) s 16(1)(c)(i); *VAD Act* (Tas) s 6(1)(a)(b); *VAD Act* (SA) s 26(1)(d)(ii); *VAD Act* (Qld) s 10(1)(a)(i); *VAD Act* (NSW) s 16(1)(d)(i)). There are, however, points of distinction regarding the definition of the UMC that require further discussion.

Under the Victorian and South Australian legislation, additional provisions have been included requiring that the UMC also be incurable (*VAD Act* (Vic) s 9(1)(d)(i); *VAD Act* (SA) s26(1)(d)(i)), and in Tasmania, the requirement of incurability is complemented by the term “irreversible” (*VAD Act*(Tas) s 6(1)(a)). The *VAD Act* (Tas) clarifies these terms, explaining that the UMC is incurable and irreversible when “there is no reasonably available treatment that can cure or reverse the [UMC] and prevent the expected death of the person” (s 6(2)(b)). This variation in terminology regarding the UMC is, however, unlikely to result in any substantive difference in practice though.

In Queensland, the Queensland Law Reform Commission specifically addressed the reasons underlying the exclusion of the term “incurable” in the eligibility criteria under the *VAD Act* (Qld) which requires that the UMC is “advanced, progressive and will cause death” (s 10(1)(a)(i)). The Queensland Law Reform Commission remarked that “it [was] not necessary for the term ‘incurable’ to be included in the eligibility criteria, as the word does not materially add to [the preferred eligibility criteria]” (Queensland Law Reform Commission (2021) 28). It was further noted that the term “advanced, progressive and will cause death” is more reflective of contemporary medical terminology and is clear in its application—that the UMC is “very serious and on a deteriorating trajectory” (Queensland Law Reform Commission (2021) 28; Government of Western Australia (2019) 34). Thus, despite the evident variation in the legislative response to defining the UMC in the individual states, it appears that the reasoning behind excluding the requirement that the UMC be incurable is not intended to have a substantial impact on eligibility to access VAD in Queensland. Therefore, it is unlikely that this will separate Queensland from the other Australian states on this point. The most significant point of difference concerning eligibility to access VAD is evident in the *VAD Act* (Tas), as exemptions from meeting the life expectancy requirement can be granted. It is not possible to engage in further discussion on this point though as the Tasmanian legislation just commenced operation.

## Conclusion

At the time of writing this Recent Developments column, VAD is only operational in Victoria, Western Australia, and Tasmania, as the other three states are still implementing the legislation. While, there is broad evidence of a consensus concerning the residency requirements and definition of the underlying medical condition to access VAD, it is evident that there are also nuances concerning these specific eligibility criteria. Despite the legislative differences considered above, it is clear that the individual Australian states have adopted a strict position concerning access to VAD; first, by ensuring that it remains an option for adults who are truly at the end of life; and second, by restricting access to VAD to persons who can establish Australian citizenship or permanent residency status—or in some jurisdictions, three continuous years of residency in Australia—in addition to specific state residency requirements.

## Footnotes

1 It is necessary to note that in 1995, the Northern Territory, Australia, enacted the *Rights of the Terminally Ill Act 1995* (NT), making it the first jurisdiction in the world to legalize voluntary assisted dying as an act of parliament. However, the NT legislation was only operational for several months after being invalidated by the Commonwealth Parliament enacting the *Euthanasia Laws Act 1997* (Cth).

2 See South Australia, *Hansard,* Legislative Council, 17 March 2021, 2958 (Minister for Health and Wellbeing).

3 In Victoria, Australia, Bills introduced into Parliament must be accompanied by a statement of compatibility with human rights and responsibilities, which is laid before the House prior to the introducing member’s second reading speech, see *Charter of Human Rights and Responsibilities Act 2006* [Vic] s 28(1), (2). In the Statement of Compatibility that accompanied the Voluntary Assisted Dying Bill 2017 [Vic], it was explained that, “this criterion is designed to … prevent people coming from outside Victoria to obtain access to voluntary assisted dying, in circumstances where such persons are unlikely to have a therapeutic relationship with a Victorian medical practitioner,” thus defending this measure on the premise of patient safety.
